# The effects of a four-week intensive scoliosis-specific exercise programme on patient-reported quality of life in adult subjects with idiopathic scoliosis: a >3 years follow-up study

**DOI:** 10.1186/1748-7161-9-S1-O69

**Published:** 2014-12-04

**Authors:** Erika Maude, Jason Black, David Glynn, Juliet Mayes, Christine Pilcher, Charlie Meekings

**Affiliations:** 1 Scoliosis SOS Clinic, London, UK; 2 Independent Statistician, London, UK

## Background

Health-related quality of life (QoL) is often reduced in adults with scoliosis [1]; therefore it is essential for any treatment they receive to address this. The Scoliosis Research Society-30 (SRS-30) questionnaire is a widely used [2], specific instrument to measure clinical outcomes in patients with scoliosis [3].

## Aim

The aim of this observational case series is to investigate previous results on whether a four-week intensive scoliosis-specific exercise programme improves patient-reported QoL in subjects with idiopathic scoliosis, by using a much larger number of participants, longer follow-up and only including adult patients.

## Design/methods

The data set was composed of 731 adult patients (578 females and 153 males) with idiopathic scoliosis and a mean age of 33 years (range 18-64 years, SD 14.68) who were treated with a four-week intensive course of scoliosis-specific physiotherapy (the ScolioGold method) between 2006 and 2013. All patients were asked to rate their QoL on their first day of treatment, at the end of their four-week course and at any subsequent check-up appointments they attended, using a modified version of the SRS-30 questionnaire (replacing ‘surgery’ with ‘treatment’). Each subset, which was analysed from the original data set, was determined by having data pre-treatment and at the relevant time point.

## Results

In the cohort analysed before and after treatment (n=512), mean total SRS-30 score increased from 3.19 (SD 0.58) to 3.60 (SD 0.47). For the cohort analysed before treatment and at >3 years (n=64), SRS-30 score increased from 3.23 (SD 0.58) to 3.69 (SD 0.44). Increases in QoL compared to pre-treatment results were found to be statistically significant (p = <0.05 using a pairwise t-test, corrected for multiple comparisons) at all time points investigated (post-treatment, 1 year, 3 years, >3 years). This was reflected in all subscales, with the exception of function.

## Conclusion

These results show the positive effect of intensive exercise methods, such as ScolioGold, on adult patients’ QoL and add to the evidence for scoliosis-specific physiotherapy. However, future research is required to establish the effects of treatment on those adults who elect not to return for check-up appointments, including those who may, or may not, have discontinued treatment.
